# Supplementary material for: The anterior insular cortex mediates higher-order threat learning and memory
Source: bioRxiv. 2026 Oct 11:2026.10.09.757763. Preprint. [Version 1] doi: 10.64898/2026.10.09.757763 (PMC13692184; doi:10.64898/2026.10.09.757763)
Supplement: Supplementary file 1 [file media-1.pdf]

Supplementary information

The anterior insular cortex mediates higher-order threat learning and memory

Ho Namkung[1], Bandhan Mukherjee[2], Josephine de Chabot[3], Sedona Lockhart[3], Lauren Guttman[3], Imad Isehak[3], Annatje Clark[3], Atsushi Saito[2], Isamu Miura[3], Koko Ishizuka[3], Hyung-Bae Kwon[1,4], and Akira Sawa[1,2,3,4,5,6*]

Departments of [1]Biomedical Engineering, [2]Pharmacology, [3]Psychiatry, [4]Neuroscience, and [5]Genetic Medicine, Johns Hopkins University School of Medicine, Baltimore, MD 21287 USA.
Department of [6]Mental Health, Johns Hopkins University Bloomberg School of Public Health, Baltimore, MD 21205, USA.

*Correspondence to: Akira Sawa, asawa1@jhmi.edu

Fig. S1

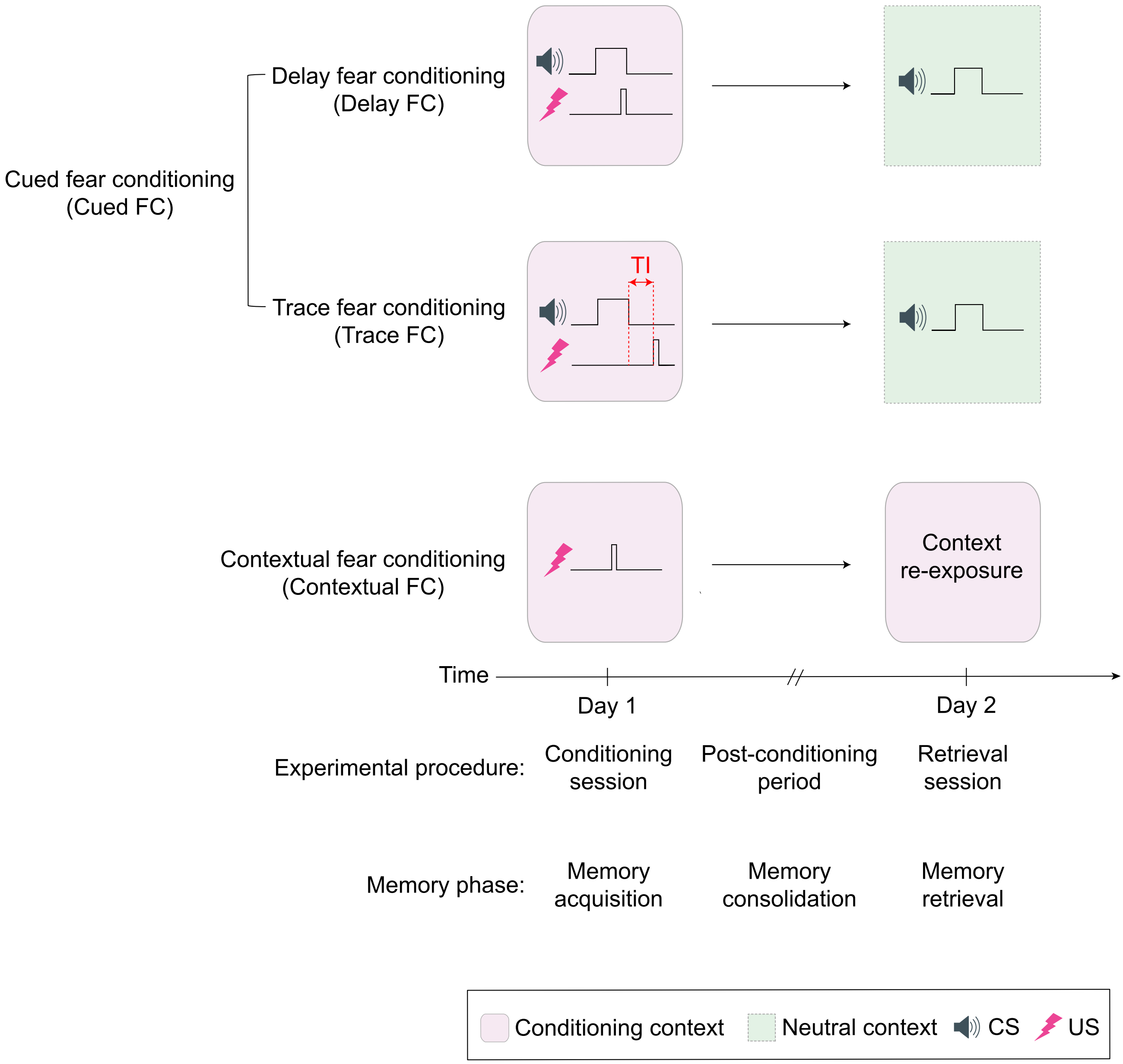

Cued fear conditioning
(Cued FC)
Delay fear conditioning
(Delay FC)
Trace fear conditioning
(Trace FC)
TI
Contextual fear conditioning
(Contextual FC)
Context
re-exposure
Time
Day 1
Day 2
Experimental procedure:
Conditioning session
Post-conditioning period
Retrieval session
Memory phase:
Memory acquisition
Memory consolidation
Memory retrieval
Conditioning context
Neutral context
CS
US

Figure S1. Schematic overview of fear conditioning paradigms and corresponding memory phases. Fear conditioning (FC) paradigms are broadly categorized into cued and contextual FC. Cued FC is further divided into delay and trace FC. In delay FC, the CS and US are temporally contiguous, whereas in trace FC, the CS and US are separated by a trace interval (TI). In contextual FC, the US is associated with the conditioning context rather than a discrete CS. Across paradigms, the conditioning session corresponds to memory acquisition, the post-conditioning period to memory consolidation, and the retrieval session to memory retrieval. During retrieval, cued fear memory is assessed by CS presentation in a neutral context, whereas contextual fear memory is assessed by re-exposure to the conditioning context. CS, conditioned stimulus; US, unconditioned stimulus; TI, trace interval.

Fig. S2

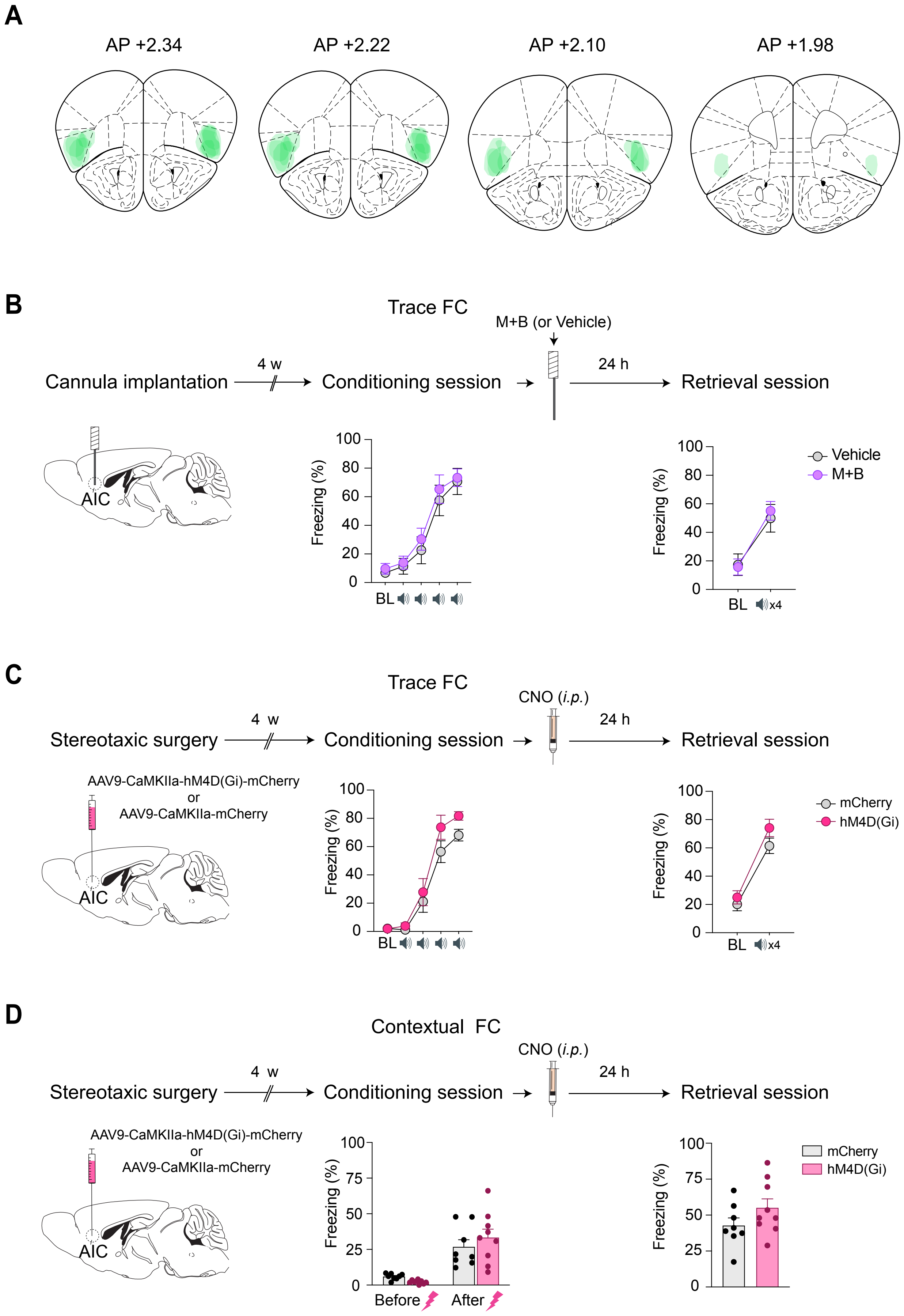
A
AP +2.34
AP +2.22
AP +2.10
AP +1.98
B
Trace FC
M+B (or Vehicle)
Cannula implantation
4 w
Conditioning session
24 h
Retrieval session
AIC
Freezing (%)
100
80
60
40
20
0
BL
x4
Vehicle
M+B
C
Trace FC
CNO (i.p.)
Stereotaxic surgery
4 w
Conditioning session
24 h
Retrieval session
AAV9-CaMKIIa-hM4D(Gi)-mCherry
or
AAV9-CaMKIIa-mCherry
AIC
mCherry
hM4D(Gi)
D
Contextual FC
CNO (i.p.)
Stereotaxic surgery
4 w
Conditioning session
24 h
Retrieval session
AAV9-CaMKIIa-hM4D(Gi)-mCherry
or
AAV9-CaMKIIa-mCherry
AIC
Freezing (%)
100
75
50
25
0
Before
After
mCherry
hM4D(Gi)

Figure S2. AIC activity is not required for fear memory consolidation in either trace or contextual FC.

(A) Coronal schematics illustrating the estimated extent of drug diffusion. It appears that the drug was primarily infused into the rostral part of the AIC, the subregion characterized by agranular cytoarchitecture.

(B) In trace FC, pharmacological inactivation of the AIC during the post-conditioning period did not disrupt fear memory acquisition. Conditioning (N_{Vehicle}=9, $N_{\mathrm{M+B}}$=9; two-way mixed ANOVA, p=0.922). Retrieval (N_{Vehicle}=9, $N_{\mathrm{M+B}}$=9; two-way mixed ANOVA, p=0.587).

(C) In trace FC, chemogenetic inhibition of AIC excitatory neurons during the post-conditioning period did not impact fear memory acquisition. Conditioning (N_{mCherry}=9, $N_{\mathrm{hM4D(Gi)}}$=9; two-way mixed ANOVA, p=0.326). Retrieval (N_{mCherry}=9, $N_{\mathrm{hM4D(Gi)}}$=9; two-way mixed ANOVA, p=0.441).

(D) In contextual FC, chemogenetic inhibition of AIC excitatory neurons during the post-conditioning period did not affect fear memory acquisition. Conditioning (N_{mCherry}=8, $N_{\mathrm{hM4D(Gi)}}$=9; two-way mixed ANOVA, p=0.212). Retrieval (N_{mCherry}=8, $N_{\mathrm{hM4D(Gi)}}$=9; two-tailed Student's t-test, p=0.156).

More detailed statistical information is in Table S1.

Fig. S3

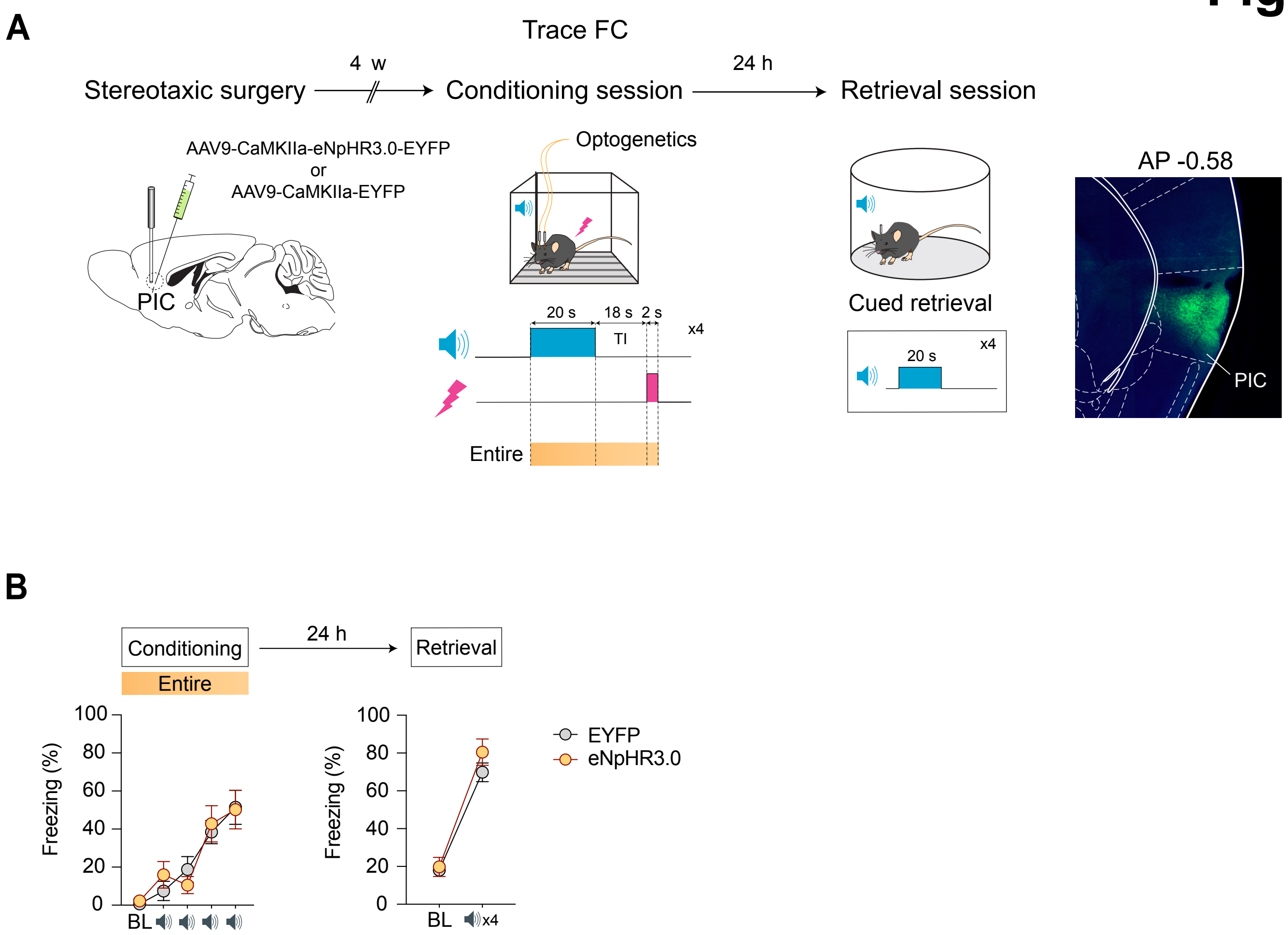
A
Trace FC
Stereotaxic surgery
4 w
Conditioning session
24 h
Retrieval session
AAV9-CaMKIIa-eNpHR3.0-EYFP
or
AAV9-CaMKIIa-EYFP
PIC
Optogenetics
20 s
18 s
2 s
TI
x4
Entire
Cued retrieval
20 s
x4
AP -0.58
PIC
B
Conditioning
24 h
Retrieval
Entire
Freezing (%)
100
80
60
40
20
0
BL
x4
EYFP
eNpHR3.0

Figure S3. Activity of PIC excitatory neurons is not required for fear memory acquisition in trace FC.

(A) Schematic of the optogenetic inhibition experiments targeting PIC excitatory neurons.

(B) Optogenetic inhibition of PIC excitatory neurons throughout the entire CS-TI-US period did not affect fear memory acquisition in trace FC. Conditioning (N_{EYFP}=10, $N_{eNpHR3.0}$=11; two-way mixed ANOVA, p=0.630). Retrieval (N_{EYFP}=10, $N_{eNpHR3.0}$=11; two-way mixed ANOVA, p=0.286).

More detailed statistical information is in Table S1.

Fig. S4

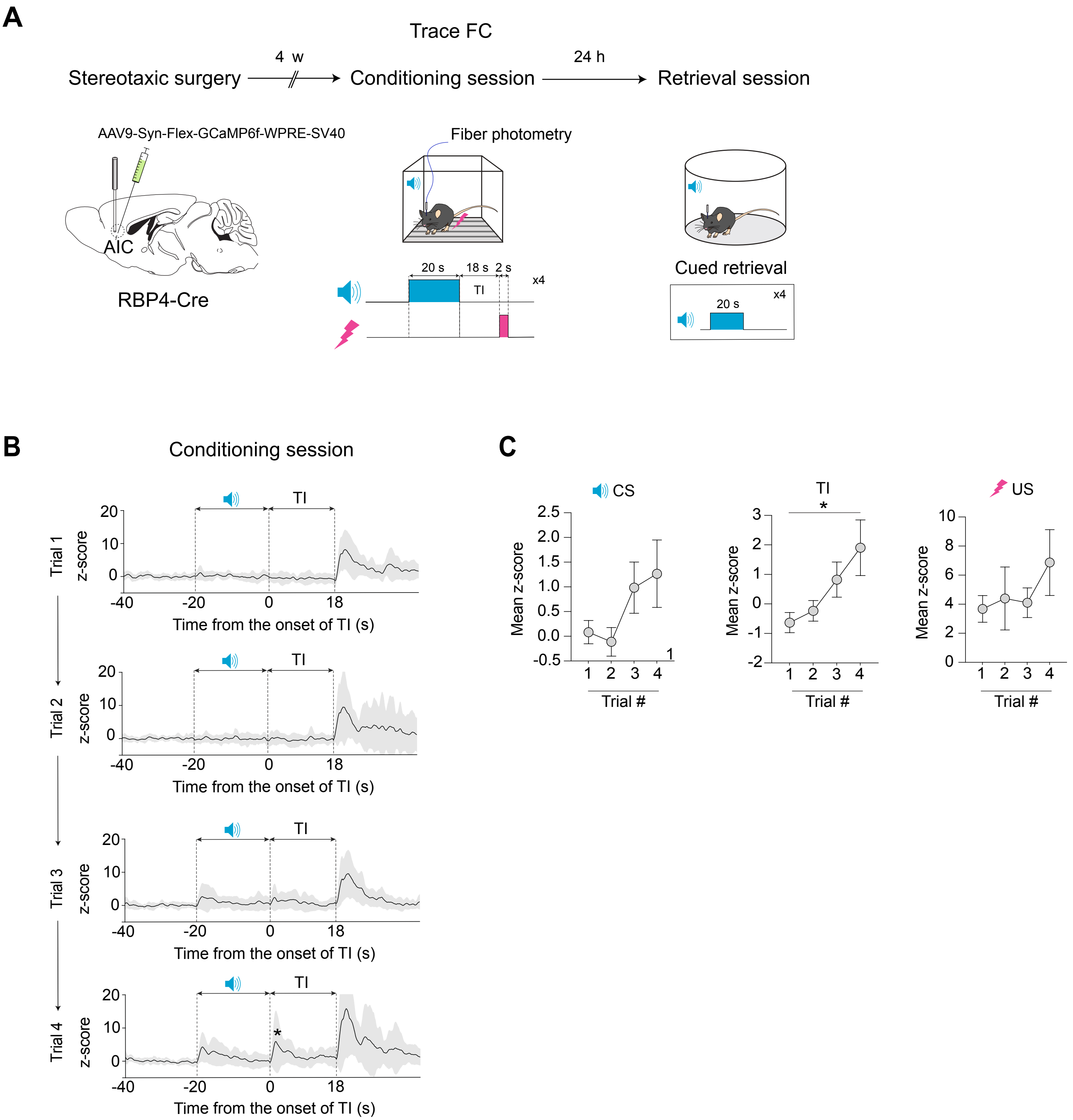
A
Trace FC
Stereotaxic surgery
4 w
Conditioning session
24 h
Retrieval session
AAV9-Syn-Flex-GCaMP6f-WPRE-SV40
AIC
RBP4-Cre
Fiber photometry
20 s
18 s
2 s
TI
x4
Cued retrieval
x4
20 s
B
Conditioning session
Trial 1
Trial 2
Trial 3
Trial 4
z-score
TI
Time from the onset of TI (s)
C
CS
TI
US
Mean z-score
Trial #

Figure S4. AIC L5-PNs show a learning-related activity at the onset of the TI that becomes increasingly prominent across the trials during the conditioning session.

(A) Schematic of the fiber photometry experiments recording population-level activity in AIC L5-PNs.

(B) Recordings revealed a transient activity (asterisk) time-locked to the onset of the TI across four CS-US pairing trials (Trial 1 - Trial4).

(C) During the conditioning session, mean activity significantly increased during the TI, but not during the CS or the US, across the trials. CS (N=10; one-way repeated measures ANOVA, p=0.092). TI (N=10; one-way repeated measures ANOVA, p=0.029). US (N=10; one-way repeated measures ANOVA, p=0.220). *$p < 0.05$, Data are expressed as mean ± SEM. More detailed statistical information is in Table S1.

Fig. S5

A

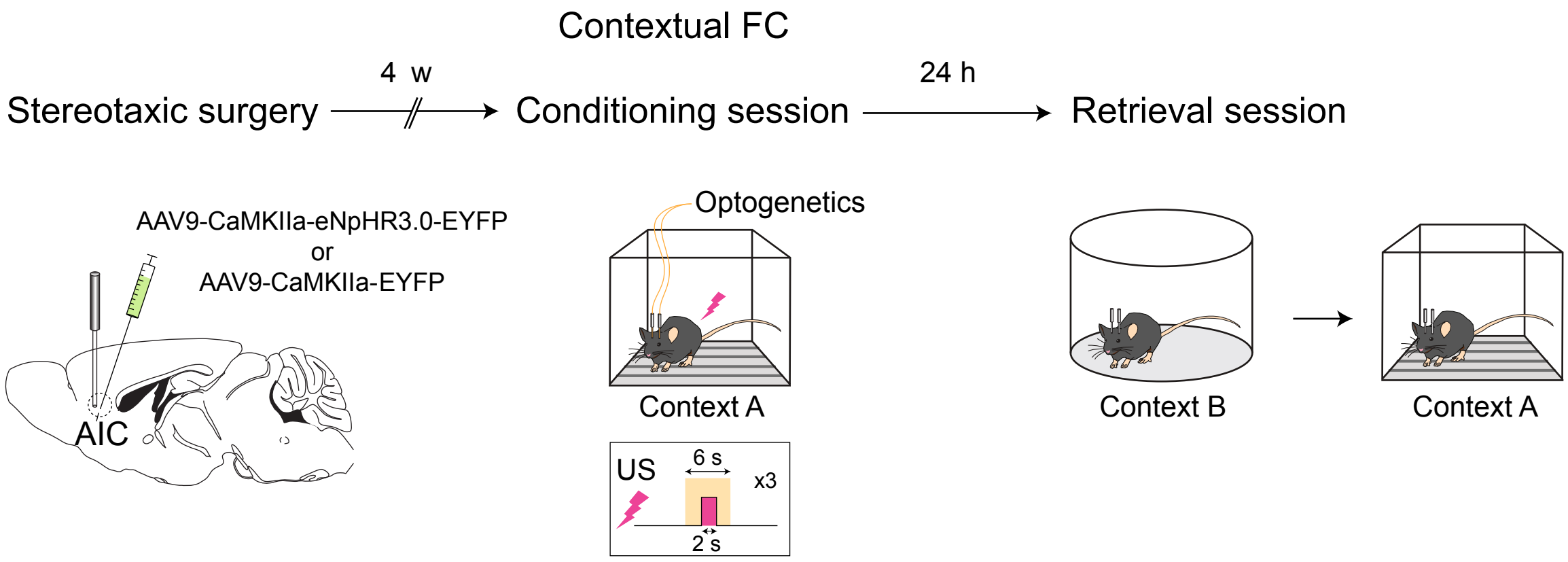
Contextual FC
Stereotaxic surgery
4 w
Conditioning session
24 h
Retrieval session
AAV9-CaMKIIa-eNpHR3.0-EYFP
or
AAV9-CaMKIIa-EYFP
AIC
Optogenetics
Context A
US
6 s
x3
2 s
Context B
Context A

B

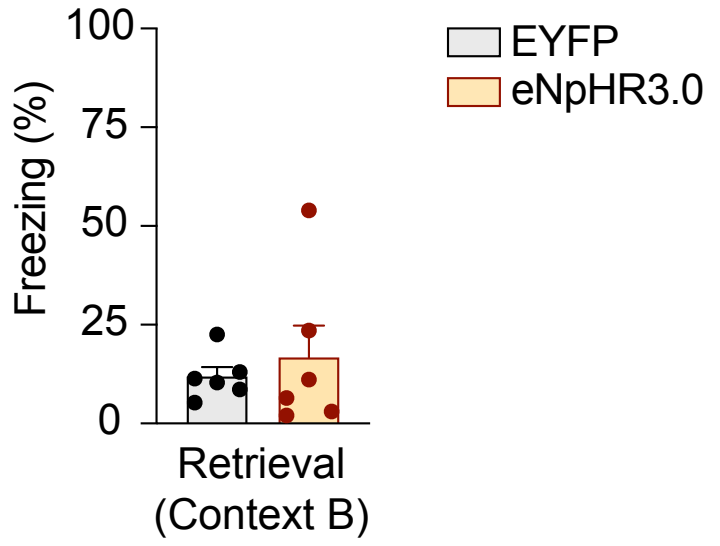
100
75
50
25
0
Freezing (%)
EYFP
eNpHR3.0
Retrieval
(Context B)

Figure S5. Optogenetic inhibition of AIC excitatory neurons during the conditioning session does not affect fear responses to a neutral context.

(A) Schematic of temporally specific optogenetic inhibition experiments during the conditioning session.

(B) Optogenetic inhibition of AIC excitatory neurons during the 6-second time windows that surround each 2-second US did not affect fear responses to a neutral context (Context B) during the retrieval session. Retrieval (N_{EYFP}=6, $N_{eNpHR3.0}$=6; two-tailed Student's *t*-test, p=0.582). More detailed statistical information is in Table S1.

Fig. S6

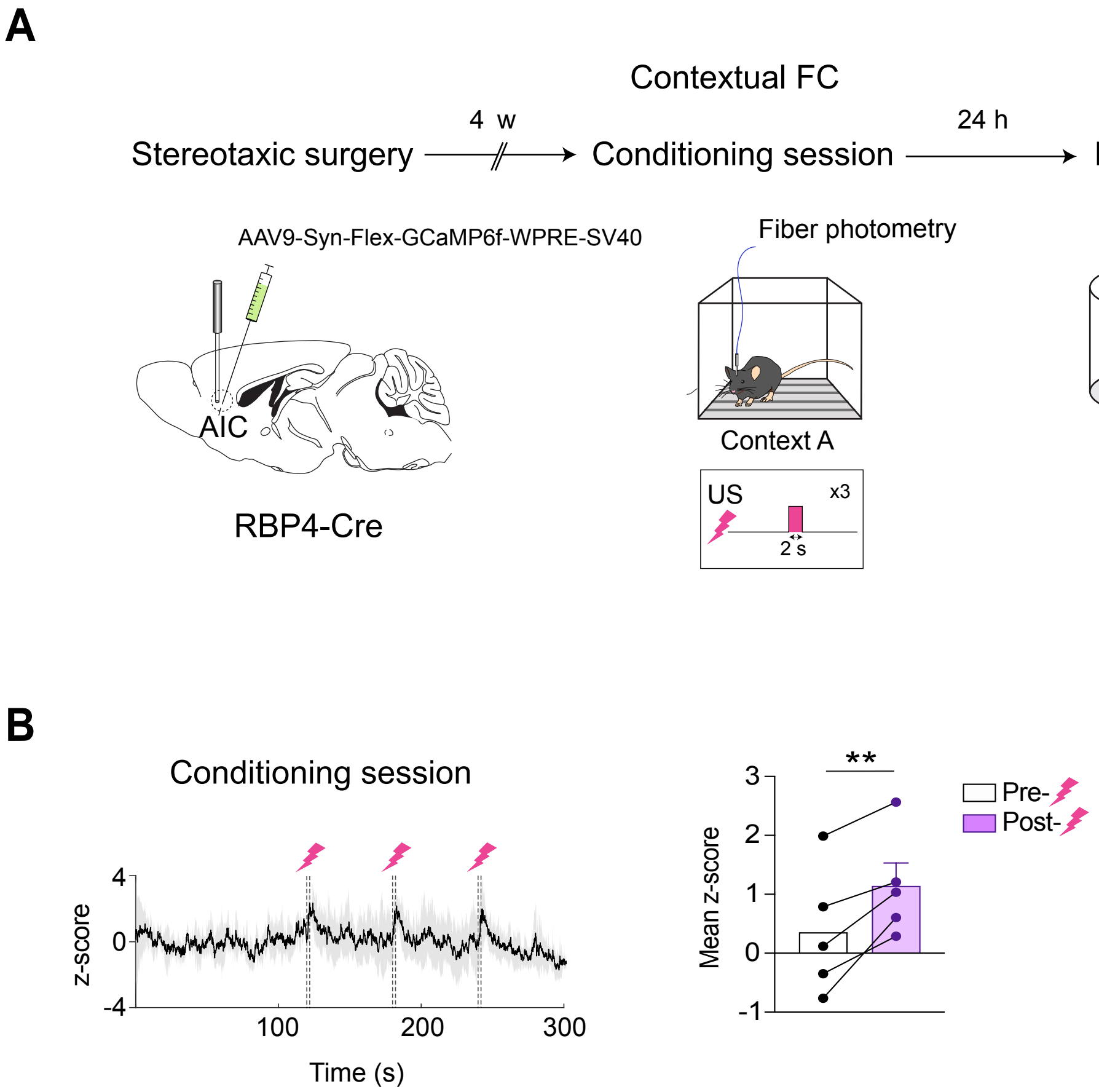
A
Contextual FC
Stereotaxic surgery
4 w
Conditioning session
24 h
Retrieval session
AAV9-Syn-Flex-GCaMP6f-WPRE-SV40
Fiber photometry
AIC
RBP4-Cre
Context A
US
x3
2 s
Context B
Context A
B
Conditioning session
z-score
Time (s)
Mean z-score
**
Pre-
Post-

Figure S6. AIC L5-PNs exhibit strong responses to the US during the conditioning session in contextual FC.

(A) Schematic of the fiber photometry experiments recording from AIC L5-PNs.

(B) Left: Population-level recordings revealed strong responses to US delivery across three context-US pairings. Right: Mean activity in the 10-s window following US onset was significantly higher than in the 10-s pre-US baseline window. mean z-score ($N = 5$; Two-tailed paired Student's t-test; $p = 0.009$). $**p < 0.01$. Data are expressed as mean ± SEM. More detailed statistical information is in Table S1.

Fig. S7

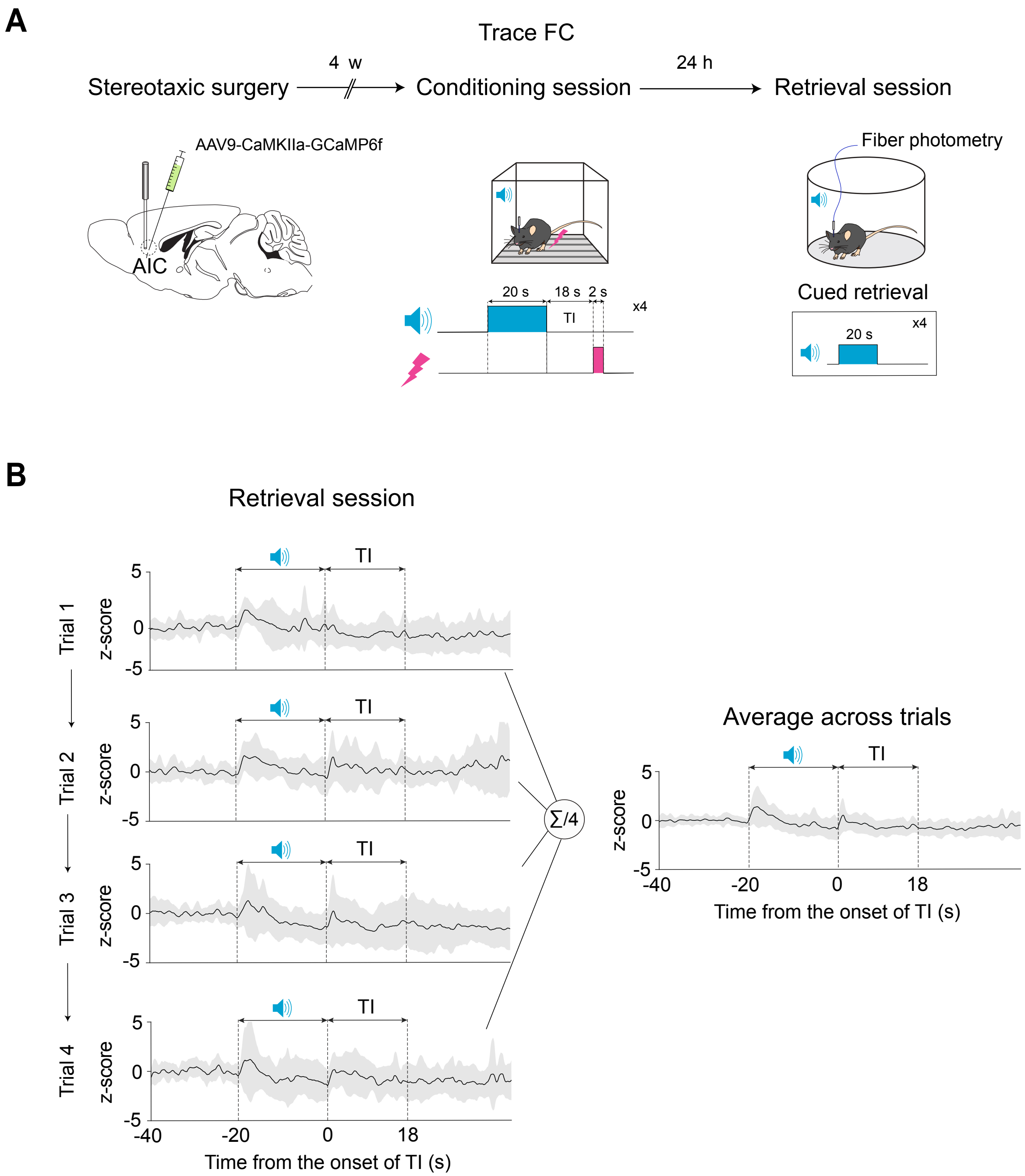

A
Trace FC
Stereotaxic surgery
4 w
Conditioning session
24 h
Retrieval session
AAV9-CaMKIIa-GCaMP6f
AIC
Fiber photometry
20 s
18 s
2 s
TI
x4
Cued retrieval
20 s
x4
B
Retrieval session
Trial 1
Trial 2
Trial 3
Trial 4
z-score
TI
Σ/4
Average across trials
Time from the onset of TI (s)
-40
-20
0
18
5
0
-5

Figure S7. During the retrieval session in trace FC, AIC excitatory neurons maintain consistent responses to the CS and the TI across trials.

(A) Schematic of the fiber photometry experiments recording from AIC excitatory neurons.

(B) Population-level recordings of AIC excitatory neurons showed their consistent responses to the CS and the TI during the retrieval session in trace FC.

Fig. S8

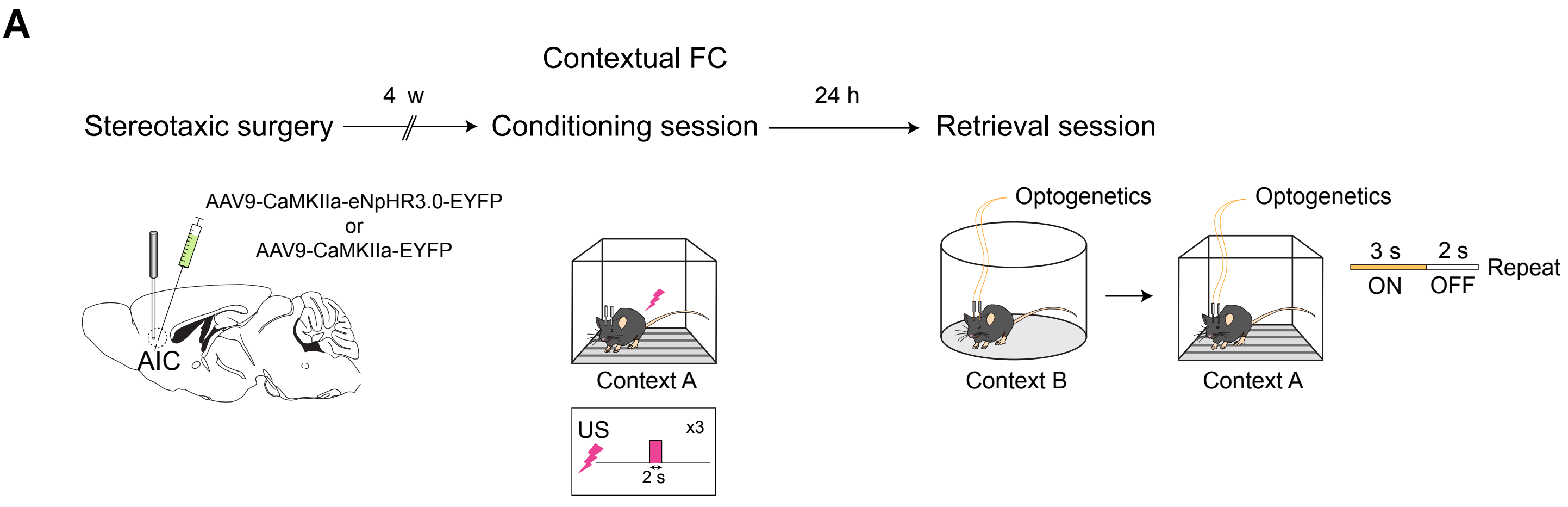

A
Contextual FC
Stereotaxic surgery
4 w
Conditioning session
24 h
Retrieval session
AAV9-CaMKIIa-eNpHR3.0-EYFP
or
AAV9-CaMKIIa-EYFP
AIC
Context A
US
x3
2 s
Optogenetics
Optogenetics
Context B
Context A
3 s
2 s
ON
OFF
Repeat

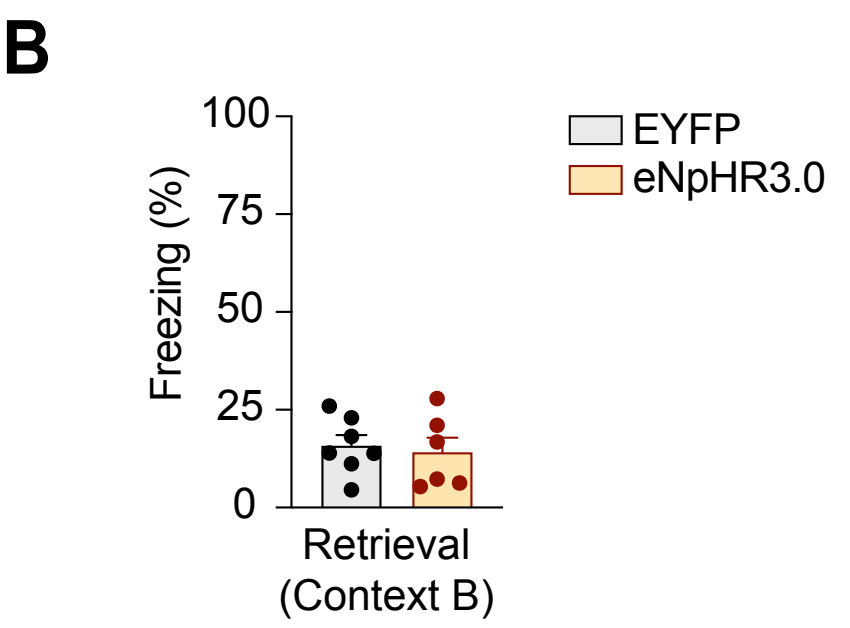

B
Freezing (%)
100
75
50
25
0
EYFP
eNpHR3.0
Retrieval
(Context B)

Figure S8. Optogenetic inhibition of AIC excitatory neurons during the retrieval session does not affect fear responses to a neutral context.

(A) Schematic of temporally specific optogenetic inhibition experiments during the retrieval session.

(B) Optogenetic inhibition of AIC excitatory neurons during the retrieval session on a 3-second ON/2-second OFF cycle did not alter fear responses to a neutral context (Context B). Retrieval (N_{EYFP}=7, $N_{\mathrm{eNpHR3.0}}$=6; two-tailed Student's *t*-test, p=0.717). More detailed statistical information is in Table S1.

Fig. S9

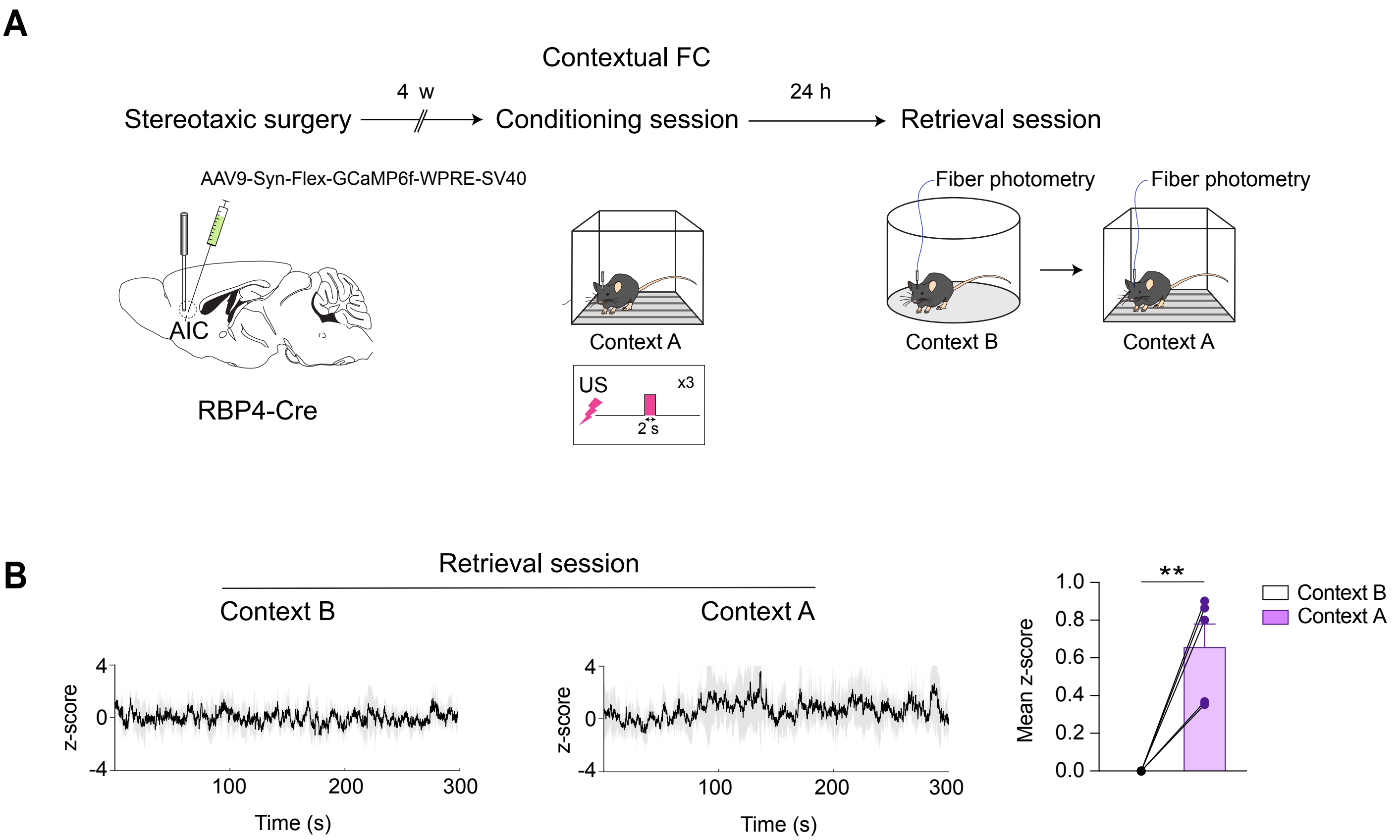
A
Contextual FC
Stereotaxic surgery
4 w
Conditioning session
24 h
Retrieval session
AAV9-Syn-Flex-GCaMP6f-WPRE-SV40
AIC
RBP4-Cre
Context A
US
x3
2 s
Fiber photometry
Fiber photometry
Context B
Context A
B
Retrieval session
Context B
Context A
z-score
4
0
-4
100
200
300
Time (s)
z-score
4
0
-4
100
200
300
Time (s)
Mean z-score
1.0
0.8
0.6
0.4
0.2
0.0
**
Context B
Context A

Figure S9. AIC L5-PNs exhibit strong responses to the conditioning context during the retrieval session.

(A) Schematic of the fiber photometry experiments recording from AIC L5-PNs.

(B) Left: During the retrieval session, AIC L5-PNs exhibit elevated, context-driven activity when mice were exposed to the conditioning context (Context A) but not to a neutral context (Context B). Right: Mean activity was significantly greater during exposure to the conditioning context compared to a neutral context. mean z-score ($N = 5$; Two-tailed one-sample t-test; $p = 0.006$). $^{**}p < 0.01$. Data are expressed as mean ± SEM. More detailed statistical information is in Table S1.

Fig. S10

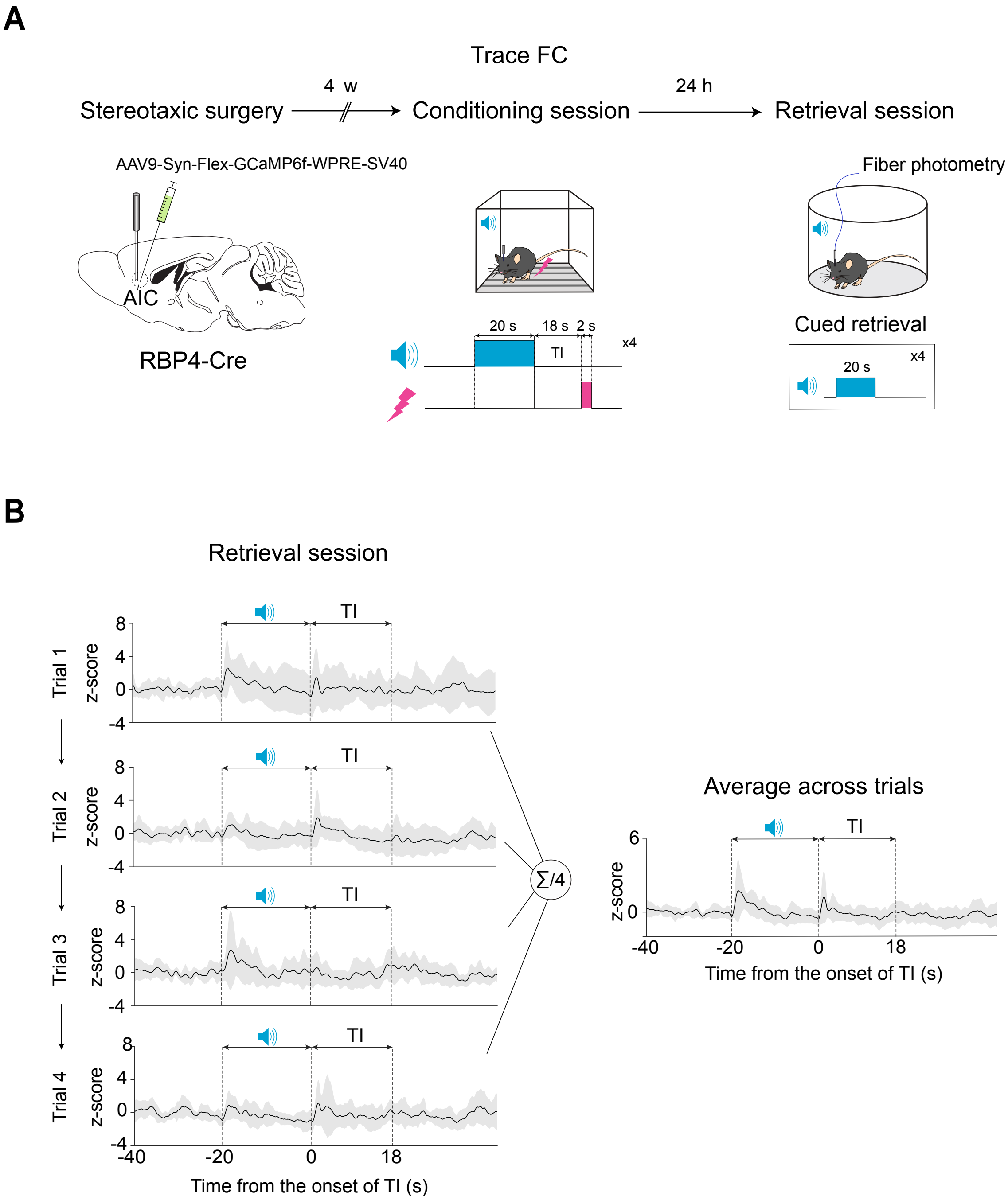
A
Trace FC
Stereotaxic surgery
4 w
Conditioning session
24 h
Retrieval session
AAV9-Syn-Flex-GCaMP6f-WPRE-SV40
AIC
RBP4-Cre
Fiber photometry
20 s
18 s
2 s
TI
x4
Cued retrieval
20 s
x4
B
Retrieval session
Trial 1
Trial 2
Trial 3
Trial 4
z-score
TI
8
4
0
-4
-40
-20
0
18
Time from the onset of TI (s)
Σ/4
Average across trials
6
0
-40
-20
0
18
Time from the onset of TI (s)

Figure S10. During the retrieval session in trace FC, AIC L5-PNs maintain consistent responses to the CS and the TI across trials.

(A) Schematic of the fiber photometry experiments recording from AIC L5-PNs.

(B) Population-level recordings of AIC L5-PNs showed their consistent responses to the CS and the TI during the retrieval session in trace FC.

Fig. S11

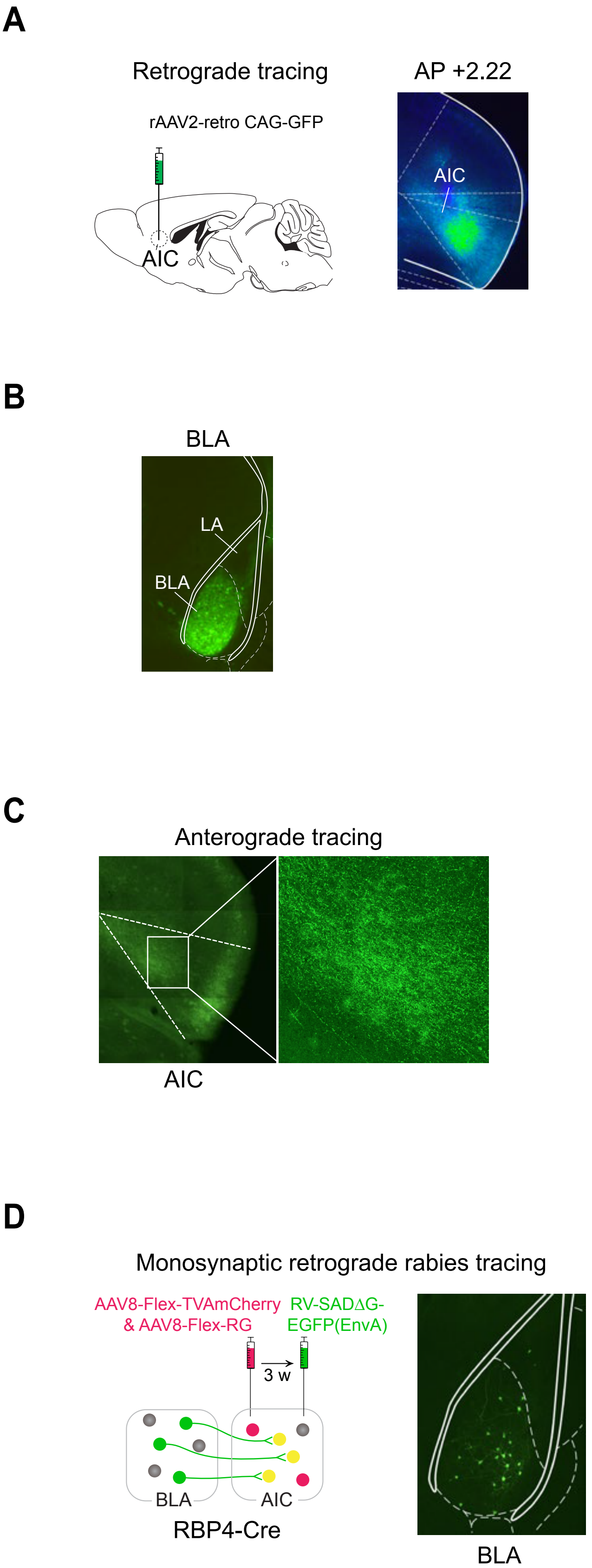
A
Retrograde tracing
rAAV2-retro CAG-GFP
AIC
AP +2.22
AIC
B
BLA
LA
BLA
C
Anterograde tracing
AIC
D
Monosynaptic retrograde rabies tracing
AAV8-Flex-TVAmCherry
& AAV8-Flex-RG
RV-SADΔG-
EGFP(EnvA)
3 w
BLA
AIC
RBP4-Cre
BLA

Figure S11. Viral tracing reveals robust inputs from the BLA to AIC L5-PNs.

(A) AAV-mediated retrograde tracing used to map afferent inputs to the AIC.

(B) Retrograde transport of GFP into the AIC revealed enriched populations of AIC-projecting neurons in the BLA.

(C) Anterograde labeling of BLA axons demonstrated dense terminal fields in layer 5.

(D) Cell type-specific, rabies virus-based retrograde tracing from AIC L5-PNs further confirmed BLA neurons that provide monosynaptic inputs.

Table S1 Statistics

Measurement	Group	Sub-group	Sample size	Average	Standard deviation	Statistical test	Statistical result	Post hoc test	Post hoc test result
Freezing (%)	Vehicle	BL	12	13.190	10.566	Two-way Mixed ANOVA with the Greenhouse-Geisser correction<br>Between-subjects factor: Drug (vehicle vs. M+B)<br>Within-subjects factor: CS	Drug: *F* (1,22)=0.122, *p* =0.731<br>CS: *F* (3.027,66.584)=58.086, *p* =1.225E-18<br>Drug*CS: *F* (3.027,66.584)=1.455, *p* =0.235	-	-
		CS1		33.200	28.584				
		CS2		42.488	24.423				
		CS3		58.967	25.055				
		CS4		72.558	16.389				
	M+B	BL	12	10.770	9.223				
		CS1		26.250	23.396				
		CS2		47.896	26.023				
		CS3		72.858	17.272				
		CS4		73.217	16.777				
Freezing (%)	Vehicle	BL	12	13.737	8.125	Two-way Mixed ANOVA<br>Between-subjects factor: Drug (vehicle vs. M+B)<br>Within-subjects factor: CS	Drug: *F* (1,22)=1.417, *p* =0.247<br>CS: *F* (1,22)=57.243, *p* =1.472E-07<br>Drug*CS: *F* (1,22)=0.703, *p* =0.411	-	-
		CS (average)		58.275	20.939				
	M+B	BL	12	12.667	7.564				
		CS (average)		48.317	25.049				
Freezing (%)	Vehicle	BL	11	8.787	14.416	Two-way Mixed ANOVA with the Greenhouse-Geisser correction<br>Between-subjects factor: Drug (vehicle vs. M+B)<br>Within-subjects factor: CS	Drug: *F* (1,22)=0.075, *p* =0.787<br>CS: *F* (2.946,64.802)=54.468, *p* =1.491E-17<br>Drug*CS: *F* (2.946,64.802)=0.596, *p* =0.617	-	-
		CS1		22.927	19.309				
		CS2		47.109	28.108				
		CS3		72.591	29.007				
		CS4		90.059	19.252				
	M+B	BL	13	12.579	17.373				
		CS1		26.915	30.502				
		CS2		44.404	40.935				
		CS3		61.065	36.351				
		CS4		85.177	21.415				
Freezing (%)	Vehicle	BL	11	9.977	12.308	Two-way Mixed ANOVA<br>Between-subjects factor: Drug (vehicle vs. M+B)<br>Within-subjects factor: CS	Drug: *F* (1,22)=3.276, *p* =0.084<br>CS: *F* (1,22)=56.336, *p* =1.674E-07<br>Drug*CS: *F* (1,22)=7.888, *p* =0.010	-	-
		CS (average)		58.439	24.438				
	M+B	BL	13	10.388	10.763				
		CS (average)		32.458	28.853				
Freezing (%)	Vehicle	BL	10	5.727	5.249	Two-way Mixed ANOVA with the Greenhouse-Geisser correction<br>Between-subjects factor: Drug (vehicle vs. M+B)<br>Within-subjects factor: CS	Drug: *F* (1,20)=0.087, *p* =0.771<br>CS: *F* (2.787,55.744)=55.414, *p* =3.224E-16<br>Drug*CS: *F* (2.787,55.744)=1.084, *p* =0.360	-	-
		CS1		19.035	26.247				
		CS2		48.360	27.738				
		CS3		66.915	17.164				
		CS4		75.020	14.545				
	M+B	BL	12	9.292	9.927				
		CS1		27.104	20.626				
		CS2		37.250	19.290				
		CS3		66.883	12.460				
		CS4		68.308	19.522				
Freezing (%)	Vehicle	BL	10	12.360	11.738	Two-way Mixed ANOVA<br>Between-subjects factor: Drug (vehicle vs. M+B)<br>Within-subjects factor: CS	Drug: *F* (1,20)=0.056, *p* =0.816<br>CS: *F* (1,20)=55.105, *p* =3.630E-07<br>Drug*CS: *F* (1,20)=1.646, *p* =0.214	-	-
		CS (average)		63.105	24.303				
	M+B	BL	12	18.020	19.495				
		CS (average)		53.809	29.537				
Freezing (%)	Vehicle	BL	10	7.456	2.781	Two-way Mixed ANOVA with the Greenhouse-Geisser correction<br>Between-subjects factor: Drug (vehicle vs. M+B)<br>Within-subjects factor: CS	Drug: *F* (1,18)=0.010, *p* =0.920<br>CS: *F* (2.105,37.899)=50.853, *p* =1.079E-11<br>Drug*CS: *F* (2.105,37.899)=2.134, *p* =0.130	-	-
		CS1		27.483	7.778				
		CS2		33.268	10.277				
		CS3		59.033	21.634				
		CS4		62.599	31.177				
	M+B	BL	10	3.981	1.685				
		CS1		14.067	6.642				
		CS2		30.583	10.777				
		CS3		59.465	31.350				
		CS4		79.701	18.816				
Freezing (%)	Vehicle	BL	10	15.471	9.550	Two-way Mixed ANOVA<br>Between-subjects factor: Drug (vehicle vs. M+B)<br>Within-subjects factor: CS	Drug: *F* (1,18)=0.566, *p* =0.462<br>CS: *F* (1,18)=88.237, *p* =2.319E-08<br>Drug*CS: *F* (1,18)=0.355, *p* =0.559	-	-
		CS (average)		70.955	16.066				
	M+B	BL	10	22.462	8.583				
		CS (average)		71.325	25.933				
Freezing (%)	EYFP	BL	10	14.0500	16.70304	Two-way Mixed ANOVA with the Greenhouse-Geisser<br>Between-subjects factor: AAV (EYFP vs. eNpHR3.0)<br>Within-subjects factor: CS	AAV: *F* (1,18)=0.256, *p* =0.619<br>CS: *F* (2.430,43.736)=13.537, *p* =8.507E-06<br>AAV*CS: *F* (2.430,43.736)=0.589, *p* =0.591	-	-
		CS1		17.6300	29.74749				
		CS2		26.9300	30.96482				
		CS3		37.4400	30.57712				
		CS4		53.4200	32.19896				
	eNpHR3.0	BL	10	7.6900	8.37675				
		CS1		7.4900	7.77695				
		CS2		21.1100	25.24561				
		CS3		46.1800	26.80571				
		CS4		47.0600	34.13780				
Freezing (%)	EYFP	BL	10	10.9500	5.39861	Two-way Mixed ANOVA<br>Between-subjects factor: AAV (EYFP vs. eNpHR3.0)<br>Within-subjects factor: CS	AAV: *F* (1,18)=8.993, *p* =0.008<br>CS: *F* (1,18)=86.416, *p* =2.713E-08<br>AAV*CS: *F* (1,18)=8.225, *p* =0.009	-	-
		CS (average)		66.4900	26.09523				
	eNpHR3.0	BL	10	7.5300	7.30389				
		CS (average)		36.8800	16.12705				

Measurement	Group	Sub-group	Sample size	Average	Standard deviation	Statistical test	Statistical result	Post hoc test	Post hoc test result
Freezing (%)	EYFP	BL	10	7.050	6.910	Two-way Mixed ANOVA with the Greenhouse-Geisser correction<br>Between-subjects factor: AAV (EYFP vs. eNpHR3.0)<br>Within-subjects factor: CS	AAV: F (1,18)=0.142, p =0.711<br>CS: F (2.572,46.294)=25.706, p =2.890E-09<br>AAV*CS: F (2.572,46.294)=0.201, p =0.868	-	-
		CS1		7.430	9.643				
		CS2		32.010	23.714				
		CS3		59.330	42.070				
		CS4		62.350	37.163				
	eNpHR3.0	BL	10	12.610	13.325				
		CS1		15.740	18.649				
		CS2		36.920	28.599				
		CS3		59.590	36.041				
		CS4		59.740	33.783				
Freezing (%)	EYFP	BL	10	11.500	16.037	Two-way Mixed ANOVA<br>Between-subjects factor: AAV (EYFP vs. eNpHR3.0)<br>Within-subjects factor: CS	AAV: F (1,18)=5.081, p =0.037<br>CS: F (1,18)=36.388, p =1.055E-05<br>AAV*CS: F (1,18)=5.919, p =0.026	-	-
		CS (average)		66.720	27.294				
	eNpHR3.0	BL	10	9.850	8.699				
		CS (average)		33.330	31.329				
Freezing (%)	EYFP	BL	11	13.782	14.590	Two-way Mixed ANOVA<br>Between-subjects factor: AAV (EYFP vs. eNpHR3.0)<br>Within-subjects factor: CS	AAV: F (1,20)=0.651, p =0.429<br>CS: F (4,80)=30.510, p =2.026E-15<br>AAV*CS: F (4,80)=0.550, p =0.700	-	-
		CS1		27.109	27.425				
		CS2		48.391	26.769				
		CS3		56.336	32.940				
		CS4		73.055	27.080				
	eNpHR3.0	BL	11	9.782	8.516				
		CS1		14.845	15.555				
		CS2		34.564	31.169				
		CS3		57.982	28.957				
		CS4		71.027	33.013				
Freezing (%)	EYFP	BL	11	21.955	23.534	Two-way Mixed ANOVA<br>Between-subjects factor: AAV (EYFP vs. eNpHR3.0)<br>Within-subjects factor: CS	AAV: F (1,20)=0.239, p =0.630<br>CS: F (1,20)=76.578, p =2.837E-08<br>AAV*CS: F (1,20)=0.170, p =0.685	-	-
		CS (average)		70.282	19.835				
	eNpHR3.0	BL	11	20.627	19.884				
		CS (average)		64.609	19.935				
Freezing (%)	EYFP	BL	11	19.073	21.776	Two-way Mixed ANOVA<br>Between-subjects factor: AAV (EYFP vs. eNpHR3.0)<br>Within-subjects factor: CS	AAV: F (1,20)=0.360, p =0.555<br>CS: F (4,80)=34.810, p =8.099E-17<br>AAV*CS: F (4,80)=0.897, p =0.470	-	-
		CS1		29.127	19.513				
		CS2		39.864	22.453				
		CS3		65.855	23.745				
		CS4		70.564	19.673				
	eNpHR3.0	BL	11	15.764	11.474				
		CS1		22.591	20.658				
		CS2		46.427	26.049				
		CS3		56.245	34.965				
		CS4		60.609	25.921				
Freezing (%)	EYFP	BL	11	18.009	19.678	Two-way Mixed ANOVA<br>Between-subjects factor: AAV (EYFP vs. eNpHR3.0)<br>Within-subjects factor: CS	AAV: F (1,20)=0.943, p =0.343<br>CS: F (1,20)=48.837, p =8.807E-07<br>AAV*CS: F (1,20)=2.470, p =0.132	-	-
		CS (average)		66.755	25.434				
	eNpHR3.0	BL	11	19.036	17.202				
		CS (average)		49.882	29.107				
Mean z-score	CS	CS1	12	-0.090	1.018	One-way Repeated Measures ANOVA<br>Repeated factor: CS	CS: F (3,33)=0.731, p =0.552		
		CS2		-0.263	1.476				
		CS3		0.125	0.688				
		CS4		0.347	1.037				
	TI	TI1		-0.852	1.366	One-way Repeated Measures ANOVA<br>Repeated factor: TI	TI: F (3,33)=3.996, p =0.016		
		TI2		-0.660	1.068				
		TI3		-0.032	0.615				
		TI4		0.549	0.997				
	US	US1		3.468	3.391	One-way Repeated Measures ANOVA<br>Repeated factor: US	US: F (3,33)=1.732, p =0.179		
		US2		2.132	2.635				
		US3		4.629	3.114				
		US4		3.705	2.742				
GFP intensity (a.u.)	Layer	L1	3	1.946	0.448	One-way ANOVA	F (3,8)=33.346, p =7.176E-05	Tukey's HSD	p (L1 vs. L2/3)=0.073,<br>p (L1 vs. L5)=7.498E-05<br>p (L1 vs. L6)=0.661<br>p (L2/3 vs. L5)=0.001<br>p (L2/3 vs. L6)=0.356<br>p (L5 vs. L6)=2.011E-04
		L2/3		9.990	3.662				
		L5		27.119	5.296				
		L6		5.156	1.886				

Measurement	Group	Sub-group	Sample size	Average	Standard deviation	Statistical test	Statistical result	Post hoc test	Post hoc test result
Freezing (%)	mCherry	BL	8	9.200	12.456	Two-way Mixed ANOVA with the Greenhouse-Geisser correction Between-subjects factor: AAV (mCherry vs. hM4D(Gi)) Within-subjects factor: CS	AAV: $F(1,14)=0.464$, $p=0.507$ CS: $F(2.720,38.084)=25.702$, $p=6.810\text{E-}09$ AAV*CS: $F(2.720,38.084)=1.550$, $p=0.220$	-	-
		CS1		21.675	20.876				
		CS2		35.113	24.283				
		CS3		72.413	32.788				
		CS4		59.675	34.681				
	hM4D(Gi)	BL	8	15.913	14.867				
		CS1		21.275	19.999				
		CS2		60.913	25.932				
		CS3		66.250	25.906				
		CS4		62.913	23.557				
Freezing (%)	mCherry	BL	8	14.125	9.097	Two-way Mixed ANOVA Between-subjects factor: AAV (mCherry vs. hM4D(Gi)) Within-subjects factor: CS	AAV: $F(1,14)=2.522$, $p=0.135$ CS: $F(1,14)=93.559$, $p=1.409\text{E-}07$ AAV*CS: $F(1,14)=5.336$, $p=0.037$	-	-
		CS (average)		56.338	9.315				
	hM4D(Gi)	BL	8	13.688	17.162				
		CS (average)		39.625	14.176				
Freezing (%)	EYFP	BL	10	18.080	14.054	Two-way Mixed ANOVA Between-subjects factor: AAV (EYFP vs. eOPN3) Within-subjects factor: CS	AAV: $F(1,18)=0.004$, $p=0.951$ CS: $F(4,72)=18.956$, $p=1.102\text{E-}10$ AAV*CS: $F(4,72)=0.544$, $p=0.704$	-	-
		CS1		21.620	19.113				
		CS2		40.470	29.681				
		CS3		60.160	27.996				
		CS4		62.740	28.363				
	eOPN3	BL	10	13.160	12.052				
		CS1		25.880	33.876				
		CS2		48.700	33.470				
		CS3		51.690	33.981				
		CS4		60.690	35.168				
Freezing (%)	EYFP	BL	10	15.570	16.852	Two-way Mixed ANOVA Between-subjects factor: AAV (EYFP vs. eOPN3) Within-subjects factor: CS	AAV: $F(1,18)=5.209$, $p=0.035$ CS: $F(1,18)=31.116$, $p=2.7041\text{E-}05$ AAV*CS: $F(1,18)=5.961$, $p=0.025$	-	-
		CS (average)		59.240	17.797				
	eOPN3	BL	10	14.070	13.418				
		CS (average)		31.150	25.546				
Freezing (%)	EYFP	BL	12	4.500	6.029	Two-way Mixed ANOVA Between-subjects factor: AAV (EYFP vs. eOPN3) Within-subjects factor: CS	AAV: $F(1,22)=0.673$, $p=0.421$ CS: $F(2.373,52.213)=17.540$, $p=3.911\text{E-}07$ AAV*CS: $F(2.373,52.213)=0.093$, $p=0.937$	-	-
		CS1		8.650	13.862				
		CS2		22.167	24.842				
		CS3		38.725	32.025				
		CS4		47.942	37.081				
	eOPN3	BL	12	12.625	16.909				
		CS1		15.192	25.618				
		CS2		29.733	33.147				
		CS3		44.758	23.982				
		CS4		49.525	27.517				
Freezing (%)	EYFP	BL	12	19.658	16.984	Two-way Mixed ANOVA Between-subjects factor: AAV (EYFP vs. eOPN3) Within-subjects factor: CS	AAV: $F(1,22)=0.421$, $p=0.523$ CS: $F(1,22)=61.694$, $p=7.994\text{E-}08$ AAV*CS: $F(1,22)=0.577$, $p=0.456$	-	-
		CS (average)		65.975	28.690				
	eOPN3	BL	12	18.792	14.839				
		CS (average)		56.942	27.575				
Freezing (%)	Vehicle	Before shock	7	2.5129	2.15366	Two-way Mixed ANOVA Between-subjects factor: Drug (vehicle vs. M+B) Within-subjects factor: Shock	Drug: $F(1,12)=0.838$, $p=0.378$ Shock: $F(1,12)=45.575$, $p=2.041\text{E-}05$ Drug*Shock: $F(1,12)=0.600$,	-	-
		After shock		33.5414	19.01110				
	M+B	Before shock	7	2.6900	4.70005				
		After shock		41.7586	16.83481				
Freezing (%)	Vehicle	-	7	44.1571	14.07520	Two-tailed Student's t-test	$t(12)=4.245$, $p=0.001$	-	-
	M+B	-	7	15.9214	10.56161				
Freezing (%)	Vehicle	Before shock	7	7.009	4.827	Two-way Mixed ANOVA Between-subjects factor: Drug (vehicle vs. M+B) Within-subjects factor: Shock	Drug: $F(1,12)=0.030$, $p=0.866$ Shock: $F(1,12)=43.969$, $p=2.426\text{E-}05$ Drug*Shock: $F(1,12)=0.079$,	-	-
		After shock		33.526	11.509				
	M+B	Before shock	7	4.880	3.421				
		After shock		33.737	22.512				
Freezing (%)	Vehicle	-	7	46.373	19.125	Two-tailed Student's t-test	$t(12)=-3.667$, $p=0.003$	-	-
	M+B	-	7	82.891	18.127				
Mean z-score	Shock	No-shock	6	-0.087	0.874	Two-tailed paired Student's t-test	$t(5)=-7.377$, $p=7.196\text{E-}04$	-	-
		Shock		2.301	1.194				
Freezing (%)	EYFP	Before shock	6	4.872	2.783	Two-way Mixed ANOVA Between-subjects factor: AAV (EYFP vs. eNpHR3.0) Within-subjects factor: Shock	AAV: $F(1,10)=0.010$, $p=0.924$ Shock: $F(1,10)=23.834$, $p=0.001$ AAV*Shock: $F(1,10)=0.058$, $p=0.814$	-	-
		After shock		32.037	13.760				
	eNpHR3.0	Before shock	6	5.650	4.683				
		After shock		30.260	20.685				
Freezing (%)	EYFP	-	6	42.273	22.483	Two-tailed Welch's t-test	$t(5.656)=2.505$, $p=0.049$	-	-
	eNpHR3.0	-	6	18.537	5.769				
Freezing (%)	mCherry	Before shock	5	7.780	10.878	Two-way Mixed ANOVA Between-subjects factor: AAV (mCherry vs. hM4D(Gi)) Within-subjects factor: Shock	AAV: $F(1,9)=0.007$, $p=0.936$ Shock: $F(1,9)=9.319$, $p=0.014$ AAV*Shock: $F(1,9)=0.239$, $p=0.636$	-	-
		After shock		20.060	15.197				
	hM4D(Gi)	Before shock	6	4.900	4.813				
		After shock		21.867	18.478				
Freezing (%)	mCherry	-	5	59.780	20.693	Two-tailed Student's t-test	$t(9)=2.592$, $p=0.029$	-	-
	hM4D(Gi)	-	6	27.500	20.471				
Freezing (%)	EYFP	Before shock	6	4.472	4.446	Two-way Mixed ANOVA Between-subjects factor: AAV (EYFP vs. eOPN3) Within-subjects factor: Shock	AAV: $F(1,10)=0.016$ $p=0.903$ Shock: $F(1,10)=43.765$, $p=5.961\text{E-}05$ AAV*Shock: $F(1,10)=0.146$,	-	-
		After shock		42.415	17.771				
	eOPN3	Before shock	6	5.690	8.965				
		After shock		39.495	22.216				
Freezing (%)	EYFP	-	6	51.828	20.529	Two-tailed Student's t-test	$t(10)=2.382$, $p=0.0385$	-	-
	eOPN3	-	6	26.650	15.779				

Measurement	Group	Sub-group	Sample size	Average	Standard deviation	Statistical test	Statistical result	Post hoc test	Post hoc test result
Mean z-score	Context	Context B	6	0.000	0.000	Two-tailed one-sample t-test	*t* (5)=6.727, *p* =1.100E-03	-	-
		Context A		0.585	0.213				
Freezing (%)	EYFP	Before shock	7	5.257	2.801	Two-way Mixed ANOVA Between-subjects factor: AAV (EYFP vs. eNpHR3.0) Within-subjects factor: Shock	AAV: *F* (1,11)=0.195, *p* =0.667 Shock: *F* (1,11)=72.240, *p* =3.656E-06 AAV*Shock: *F* (1,11)=0.545,	-	-
		After shock		40.886	14.267				
	eNpHR3.0	Before shock	6	5.667	5.322				-
		After shock		35.600	19.042				
Freezing (%)	EYFP	-	7	43.400	12.555	Two-tailed Student's *t* -test	*t* (11)=-2.563, *p* =0.026	-	-
	eNpHR3.0	-	6	66.650	19.895				
Freezing (%)	mCherry	Before shock	5	3.750	4.024	Two-way Mixed ANOVA Between-subjects factor: AAV (mCherry vs. hM4D(Gi)) Within-subjects factor: Shock	AAV: *F* (1,8)=0.456, *p* =0.519 Shock: *F* (1,8)=29.971, *p* =0.001 AAV*Shock: *F* (1,8)=0.536, *p* =0.485	-	-
		After shock		31.690	18.943				
	hM4D(Gi)	Before shock	5	10.994	6.217				
		After shock		32.344	11.453				
Freezing (%)	mCherry	-	5	57.048	13.858	Two-tailed Student's t-test	*t* (8)=-2.355, *p* =0.046	-	-
	hM4D(Gi)	-	5	77.282	13.304				
Freezing (%)	EYFP	Before shock	6	3.967	1.208	Two-way Mixed ANOVA Between-subjects factor: AAV (EYFP vs. eOPN3) Within-subjects factor: Shock	AAV: *F* (1,10)=1.137 *p* =0.311 Shock: *F* (1,10)=104.703, *p* =1.287E-06 AAV*Shock: *F* (1,10)=0.174,	-	-
		After shock		44.983	16.618				
	eOPN3	Before shock	6	10.600	7.333				
		After shock		48.400	10.637				
Freezing (%)	EYFP	-	6	52.883	16.623	Two-tailed Student's t-test	*t* (10)=-2.399, *p* =0.037	-	-
	eOPN3	-	6	75.600	16.173				
Cell proportion (%)		Conditioning-only	4	0.339	0.066	One-way ANOVA	*F* (2,9)=38.649, *p* =3.820×10^{-5}	Tukey's HSD	*p* (Conditioning-only vs. overlap)=0.003, *p* (Conditioning-only vs. Retrieval-only)=0.006 *p* (overlap vs. Retrieval-only)=2.762E-05
		Overlap		0.081	0.060				
		Retrieval-only		0.580	0.109				
Freezing (%)	EYFP	Before shock	5	16.498	12.833	Two-way Mixed ANOVA Between-subjects factor: AAV (EYFP vs. NpHR) Within-subjects factor: Shock	AAV: *F* (1,9)=0.375, *p* =0.555 Shock: *F* (1,9)=63.265, *p* =2.318E-05 AAV*Shock: *F* (1,9)=0.008, *p* =0.931	-	-
		After shock		51.016	11.676				
	NpHR	Before shock	6	13.573	12.818				
		After shock		47.327	7.858				
Freezing (%)	EYFP	-	5	46.884	9.108	Two-tailed Student's t-test	*t* (9)=-1.624, *p* =0.139	-	-
	NpHR	-	6	55.295	8.081				
Freezing (%)	Vehicle	BL	9	6.818	7.328	Two-way Mixed ANOVA with the Greenhouse-Geisser correction Between-subjects factor: Drug (vehicle vs. M+B) Within-subjects factor: CS	Drug: *F* (1,16)=0.605, *p* =0.448 CS: *F* (2.701,43.215)=61.384, *p* =4.988E-15 Drug*CS: *F* (2.701,43.215)=0.138, *p* =0.922	-	-
		CS1		11.427	16.603				
		CS2		22.630	28.276				
		CS3		57.445	31.985				
		CS4		73.536	21.407				
	M+B	BL	9	9.577	11.263				
		CS1		13.908	13.613				
		CS2		30.296	23.107				
		CS3		65.352	29.803				
		CS4		81.740	19.372				
Freezing (%)	Vehicle	BL	9	17.351	22.680	Two-way Mixed ANOVA Between-subjects factor: Drug (vehicle vs. M+B) Within-subjects factor: CS	Drug: *F* (1,16)=0.042, *p* =0.840 CS: *F* (1,16)=35.862, *p* =1.893E-05 Drug*CS: *F* (1,16)=0.323, *p* =0.587	-	-
		CS (average)		49.890	28.935				
	M+B	BL	9	15.732	16.860				
		CS (average)		55.093	19.651				
Freezing (%)	mCherry	BL	9	2.124	3.217	Two-way Mixed ANOVA with the Greenhouse-Geisser correction Between-subjects factor: AAV (mCherry vs. hM4D(Gi)) Within-subjects factor: CS	AAV: *F* (1,16)=2.446, *p* =0.137 CS: *F* (2.030,32.481)=103.014, *p* =7.381E-15 AAV*CS: *F* (2.030,32.481)=1.161, *p* =0.326	-	-
		CS1		1.223	2.433				
		CS2		21.168	22.745				
		CS3		56.466	23.160				
		CS4		68.148	12.239				
	hM4D(Gi)	BL	9	1.961	3.445				
		CS1		3.946	6.412				
		CS2		27.837	28.815				
		CS3		73.762	25.607				
		CS4		81.724	9.058				
Freezing (%)	mCherry	BL	9	20.109	13.760	Two-way Mixed ANOVA Between-subjects factor: AAV (mCherry vs. hM4D(Gi)) Within-subjects factor: CS	AAV: *F* (1,16)=2.482, *p* =0.135 CS: *F* (1,16)=84.577, *p* =8.694E-08 AAV*CS: *F* (1,16)=0.625, *p* =0.441	-	-
		CS (average)		61.460	16.433				
	hM4D(Gi)	BL	9	24.994	14.037				
		CS (average)		74.121	18.369				
Freezing (%)	mCherry	Before shock	8	5.936	2.146	Two-way Mixed ANOVA Between-subjects factor: AAV (mCherry vs. hM4D(Gi)) Within-subjects factor: Shock	AAV: *F* (1,15)=0.119, *p* =0.735 Shock: *F* (1,15)=44.493, *p* =7.476E-06 AAV*Shock: *F* (1,15)=1.703,	-	-
		After shock		26.809	14.174				
	hM4D(Gi)	Before shock	9	2.250	1.271				
		After shock		33.276	17.802				
Freezing (%)	mCherry	-	8	42.743	14.882	Two-tailed Student's t-test	*t* (15)=-1.493, *p* =0.156	-	-
	hM4D(Gi)	-	9	55.068	18.644				

Measurement	Group	Sub-group	Sample size	Average	Standard deviation	Statistical test	Statistical result	Post hoc test	Post hoc test result
Freezing (%)	EYFP	BL	10	0.660	0.992	Two-way Mixed ANOVA<br>Between-subjects factor: AAV (EYFP vs. eNpHR3.0)<br>Within-subjects factor: CS	AAV: *F* (1,19)=0.024, *p* =0.880<br>CS: *F* (4,76)=28.512, *p* =1.781E-14<br>AAV*CS: *F* (4,76)=0.648, *p* =0.630	-	-
		CS1		7.480	15.881				
		CS2		18.790	21.362				
		CS3		38.400	19.203				
		CS4		51.440	28.215				
	eNpHR3.0	BL	11	2.218	3.893				
		CS1		16.000	23.163				
		CS2		10.564	14.692				
		CS3		42.791	31.489				
		CS4		50.236	33.674				
Freezing (%)	EYFP	BL	10	18.090	9.099	Two-way Mixed ANOVA<br>Between-subjects factor: AAV (EYFP vs. eNpHR3.0)<br>Within-subjects factor: CS	AAV: *F* (1,19)=0.972, *p* =0.337<br>CS: *F* (1,19)=192.685, *p* =2.135E-11<br>AAV*CS: *F* (1,19)=1.205, *p* =0.286	-	-
		CS (average)		69.840	15.625				
	eNpHR3.0	BL	11	19.818	16.727				
		CS (average)		80.455	23.076				
Mean z-score	CS	CS1	10	0.083	0.745	One-way Repeated Measures ANOVA<br>Repeated factor: CS	CS: *F* (3,27)=2.374, *p* =0.092	-	-
		CS2		-0.113	0.914				
		CS3		0.984	1.642				
		CS4		1.267	2.154				
	TI	TI1		-0.632	1.084	One-way Repeated Measures ANOVA<br>Repeated factor: TI	TI: *F* (3,27)=3.505, *p* =0.029	-	-
		TI2		-0.232	1.099				
		TI3		0.824	1.883				
		TI4		1.904	2.978				
	US	US1		3.681	2.897	One-way Repeated Measures ANOVA<br>Repeated factor: US	US: *F* (3,27)=1.569, *p* =0.220	-	-
		US2		4.397	6.857				
		US3		4.104	3.230				
		US4		6.868	7.159				
Freezing (%)	EYFP	-	6	11.877	5.876	Two-tailed Student's *t* -test	*t* (10)=-0.569, *p* =0.582	-	-
	eNpHR3.0	-	6	16.683	19.847				
Mean z-score	Shock	No-shock	5	0.3564	1.08057	Two-tailed paired Student's t-test	*t* (4)=-4.685, *p* =0.009	-	-
		Shock		1.1405	0.87346				
Freezing (%)	EYFP	-	7	15.823	7.237	Two-tailed Student's *t* -test	*t* (11)=0.372, *p* =0.717	-	-
	eNpHR3.0	-	6	14.120	9.271				
Mean z-score	Context	Context B	5	0.0000	0.00000	Two-tailed one-sample t-test	*t* (4)=5.364, *p* =0.006	-	-
		Context A		0.6573	0.27399				
